# A Web-Based Tool to Report Adverse Drug Reactions by Community Pharmacists in Australia: Usability Testing Study

**DOI:** 10.2196/48976

**Published:** 2023-09-29

**Authors:** Joel Fossouo Tagne, Reginald Amin Yakob, Rachael Mcdonald, Nilmini Wickramasinghe

**Affiliations:** 1 School of Health Sciences and Biostatistics Swinburne University of Technology Melbourne Australia; 2 Centre for Health Analytics Murdoch Children's Research Institute Health Informatics Melbourne Australia; 3 Pharmaceutical Society of Australia Sydney Australia; 4 MedTechVic Swinburne University of Technology Melbourne Australia; 5 Department of Nursing and Allied Health, Occupational Therapy Swinburne University of Technology Melbourne Australia; 6 Iverson Health Innovation Research Institute Swinburne University of Technology Melbourne Australia; 7 Epworth Healthcare Melbourne Australia; 8 School of Computing, Engineering & Mathematical Sciences La Trobe University Melbourne Australia

**Keywords:** ADR, adverse drug reaction, pharmacovigilance, community pharmacy, digital health evaluation, usability testing

## Abstract

**Background:**

Adverse drug reactions (ADRs) are unintended and harmful events associated with medication use. Despite their significance in postmarketing surveillance, quality improvement, and drug safety research, ADRs are vastly underreported. Enhanced digital-based communication of ADR information to regulators and among care providers could significantly improve patient safety.

**Objective:**

This paper presents a usability evaluation of the commercially available GuildCare Adverse Event Recording system, a web-based ADR reporting system widely used by community pharmacists (CPs) in Australia.

**Methods:**

We developed a structured interview protocol encompassing remote observation, think-aloud moderating techniques, and retrospective questioning to gauge the overall user experience, complemented by the System Usability Scale (SUS) assessment. Thematic analysis was used to analyze field notes from the interviews.

**Results:**

A total of 7 CPs participated in the study, who perceived the system to have above-average usability (SUS score of 68.57). Nonetheless, the structured approach to usability testing unveiled specific functional and user interpretation issues, such as unnecessary information, lack of system clarity, and redundant data fields—critical insights not captured by the SUS results. Design elements like drop-down menus, free-text entry, checkboxes, and prefilled or auto-populated data fields were perceived as useful for enhancing system navigation and facilitating ADR reporting.

**Conclusions:**

The user-centric design of technology solutions, like the one discussed herein, is crucial to meeting CPs’ information needs and ensuring effective ADR reporting. Developers should adopt a structured approach to usability testing during the developmental phase to address identified issues comprehensively. Such a methodological approach may promote the adoption of ADR reporting systems by CPs and ultimately enhance patient safety.

## Introduction

### Overview

Patients who regularly use medications may experience adverse drug reactions (ADRs), causing increased chances of morbidity and mortality [1]. In Australia, the primary mechanism for pharmacovigilance (PV) is the spontaneous reporting of ADRs, including the postmarketing passive surveillance of licensed medications or vaccines. According to the World Health Organization (WHO), PV is the “science and activities relating to the detection, assessment, understanding, and prevention of adverse events or any other drug-related problem” [2,3]. Worldwide, ADR-related hospital admissions range from 3.6% to 15.6% [4,5]. In Australia, ADR-related hospital admissions are estimated at 7.2% to 11% [1]. A study showed that up to 51% of ADRs were deemed preventable, leading to an increase in the length of hospital stay, that is, from 8 to 20 days [6]. Medication-related problems account for approximately AU $1.4 billion (US $950,649) per annum, that is, 15% of the total Australian Pharmaceutical Benefits Scheme [7].

Despite the significant burden that ADRs pose on patients and the health care system, ADRs are often not documented by clinicians or communicated to allied health, jurisdictional public health, and federal regulatory authorities [8,9]. Drug safety reporting is an essential mechanism for early detection of untoward reactions to medications within health care systems [10]. It is essential for information regarding ADRs to be shared among health care professionals for learning purposes and patient safety [3,11]. The challenges of drug safety reporting include attitudes of reporters, such as motivation and implicated errors, lack of time or financial incentive, and problems with reporting systems [12-14]. Therefore, medication-related problems can be potentially avoided by improving health care systems and medication supply practices [15].

A 2020 systematic review of interventions to improve ADR reporting concluded that there was scope to include community pharmacists (CPs) to improve ADR reporting [12]. This is also consistent with other studies [16,17]. CPs are available in community pharmacies and are well situated to report ADRs from patients [12]. For instance, in Australian capital cities, 97% of consumers are located within a 2.5 km distance from a pharmacy, while 65% in regional or remote areas [18].

### ADR Reporting Information Systems

In a companion study, we interviewed CPs in Australia to identify barriers and facilitators to ADR reporting (the detailed methods and results of the study are published separately) [19]. On the basis of our results, we theorized that although enticements and enforcements may encourage behavioral changes toward ADR reporting, improving workflow practices and electronic ADR reporting systems may also achieve the same. These are consistent with other studies [12,20,21].

The reporting system is a modifiable category, and systems that are not suited to capture the complex nature of ADRs or adapted to a clinician’s workflow may not be used [21]. A reporting system that is laborious, along with a lack of education on ADR reporting, nonsupportive management, and a lack of feedback, discourages reporting [17]. Designing improved and effective ADR reporting technologies will require an understanding of the barriers to ADR reporting [22].

The uptake of electronic systems allows ADR reporting to be integrated into point-of-care documentation and reporting [23]. If reporting software with auto-population features could be integrated into dispensing systems, patient-level alerts may likewise be generated from the system to prevent reexposure and improve patient safety [24,25].

While integrating reporting systems into pharmacists’ dispensing software presents an opportunity for patient-level alerts, it is important to note that different pharmacies may use different types of dispensing software or professional service programs. Therefore, it is necessary for regulators or software vendors to develop uniform reporting and surveillance systems that are available nationally and capable of integrating with different pharmacy dispensing programs. To our knowledge, 2 PV systems currently exist in Australian community pharmacies [17,26]. These include the GuildCare Adverse Events Recording module, which is a web-based system that is linked to the community pharmacy dispensing software and integrates directly into the Therapeutic Goods Administration (TGA) ADR web service [17]. The second system is SmartVax, a vaccine safety surveillance system that is integrated into a cloud-based community pharmacy software system called MedAdvisor [27]. SmartVax was linked with pharmacy data in response to the COVID-19 pandemic and automatically reports immunizations administered directly to the Australian Immunisation Register [27].

Poor ADR documentation and a lack of data communication between care providers and across health care settings are major roadblocks to PV [24]. This is consistent with our 2023 scoping review, which identified that there is substantial interinstitutional variability in the standards of ADR reporting among individual primary health care facilities in Australia [28]. This further suggests that improving health care systems and medication supply practices may reduce reexposure to harmful drugs [15]. Nevertheless, a systematic review of adverse event reporting systems found wide variation in the variety and type of data collected [29]. Furthermore, these reporting systems did not report pilot-testing electronic fields to ensure that there was succinct, user-friendly, relevant, and correct interpretation of fields by care providers before their implementation [25,29]. If ADR reporting systems can be designed to meet the documentation and communication needs of the CPs who recognize adverse drug events at the point of care, CPs may be more willing to document and report [9].

During the evaluation process, many developers in the health care setting may rely on practical tools such as heuristic checklists, the time limitations of end users, questionnaires, surveys, focus groups, and interviews to assess system usability [30]. While these may shed light on the end user perceptions of usefulness, satisfaction, and implementation feasibility, a usability study involving real-time observation of users completing specified tasks typically provides added insight into the quality of user interaction with a given system within the intended setting [30,31]. Implementing new systems without pilot testing and refining may fall short of their expected goals due to systems’ architecture constraints or design failures that could have been identified and resolved before their final build [30]. In PV, usability is important to consider when assessing reporting systems. Furthermore, if PV reporting systems are cumbersome or disconnected from the normal workflow of CPs, ADR reporting can be hampered. If the task of writing an ADR report is sufficiently unpleasant, confusing, or difficult, the immediate patient care-related activities may supersede the data needs of regulators in ADR monitoring [9,32].

In this paper, we present the findings of a usability evaluation of a commercially available adverse event reporting system in Australia. This study involved real-time observation to evaluate end users’ (CPs) ability to navigate the system and generate an ADR report. The results of the study may be useful for improving or designing new ADR reporting systems available to CPs, which could stimulate reporting and improve patient safety. Furthermore, this paper may also provide an example of how to test usability before the implementation of newly designed health information systems for primary care. To our knowledge, no previous study has assessed the usability of an adverse event reporting system (dashboard) for the purposes of PV among CPs in Australia.

## Methods

### Overview

This study forms part of a larger study focused on assessing the impact of IT on facilitating the reporting of ADRs by CPs in Australia [10,19,28,33]. A qualitative study with semistructured interviews was conducted with 7 CPs working in community pharmacies across Victoria, Australia, between June 2022 and August 2022. A pharmacist with experience in community pharmacy and a biomedical engineer with experience in digital health were involved in the design and conduct of the study.

### Participants

Purposive sampling was used to select eligible participants working in community pharmacies listed on the Pharmacy Guild of Australia and Health direct website. Participants agreed to conduct a 25- to 60-minute recorded web-based interview while sharing their screen. Participants had no previous experience with the ADR recording module; they were provided with a user manual at least a day before the interview.

### Interview Protocol

Designed to evaluate both usefulness and satisfaction, the interview protocol leveraged think-aloud moderating techniques (assessing usefulness), retrospective questioning about user satisfaction, and administration of the System Usability Scale (SUS; assessing satisfaction). Usability testing relied on participants’ verbal communication and remote observation through screen sharing [34]. During the interview, participants were directed to complete an ADR report scenario using a semistructured interview protocol.

### Testing Procedure

**Figure 1:**
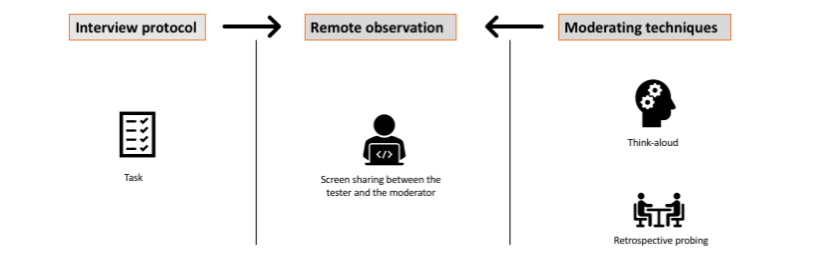
The process involved during the usability testing.

Tasks (ADR reporting) were developed by the team and revised for clarity and simplicity to establish whether the system’s features worked as intended (eg, buttons, links, or drop-down menus) and whether participants could derive appropriate meaning from the display (eg, identifying how to add the suspected medication). These tasks were highly specific to the reporting system and allowed the participant to explore the range of functions available for the purpose of completing an ADR report, including collecting patient data, adding suspected medication, recording the reaction details, and generating a report. The think-aloud technique where participants verbalize what they are thinking, doing, seeing, or feeling, while they complete a specific task or set of tasks, has been shown to generate rich data on nonobservable cognitive aspects of a design interface [34]. After completing the ADR report, participants were then tasked with completing the SUS to provide information about their experiences with the system. Upon completing the SUS, participants were then asked more general questions about user satisfaction. One member of the team (JFT) conducted aspects of the interview, including generating field notes of written documentation of participants’ observed behaviors and transcription of participants’ statements provided during the retrospective interview audio recordings. A summary of our usability testing approach can be seen in Figure 1.

### Web-Based Reporting Tool

The GuildCare Adverse Event Recording program is a web-based tool designed to enable CPs with the ability to record adverse events at the point of care with all required information and submit them to the TGA and, where necessary, to a patient’s medical practitioner (Multimedia Appendix 1) [17,35]. The web-based tool is designed to submit adverse drug events related to both medicines and vaccinations to the TGA [36].

### Data Analysis

#### Usefulness

System usefulness was assessed by analyzing participant data (n=7) recorded as they interacted with the system during the evaluation session and providing feedback regarding the system [31]. For example, we recorded whether the participants completed each task with ease (without assistance), with difficulty (requiring assistance from the researcher to complete the task), or failed to complete it.

Thematic analysis began once interviews were completed using NVivo 12 (Lumivero) and was performed by 2 members of the team. Initially, open codes were generated inductively. Following the initial coding of transcripts, preliminary themes that captured information relevant to the research questions were generated. This process involved identifying patterns within the data: recurring ideas, perspectives, and descriptions that depicted each participant’s context and perspective. The final analysis for this study focused on the key themes generated from the interview and field notes. Data concordance was verified by NW and RM, researchers with extensive experience in public and digital health research. The key themes were discussed among the research team, which included pharmacists (JFT and RAY) and an engineer. Interviews concluded when no additional themes relating to the research question could be found.

#### Satisfaction

This was evaluated using a validated questionnaire, the SUS [31]. The SUS is a flexible questionnaire designed to assess any technology and is relatively quick and easy to complete [15,37]. It consists of 10 statements that are scored on a 5-point scale (1=strongly disagree to 5=strongly agree), with the final scores (after transformation of the scores) ranging from 0 to 100 [38]. A higher score may indicate better usability. As a general rule, a system that has a score above 68 has acceptable usability; a lower score means that the system needs more scrutiny and continued improvement (Multimedia Appendices 2 and 3) [15]. To calculate the SUS score, the total score for all odd and even number questions (1, 3, 5, 7, 9 and 2, 4, 6, 8, 10, respectively) was added. The scale of odd questions was then deducted by 1, whereas for even questions, it was deducted by 5. The final SUS score calculation is done by summing the modified scale and multiplying it by 2.5 to get a score in the range of 0 to 100 [15].

### Ethical Considerations

Before conducting the interviews, all participants provided informed written consent to participate in the study and were advised that the information provided, although deidentified, could be used for publication. Participants’ demographic data were collected using a self-administered questionnaire attached to the consent form. All procedures were in accordance with Australia’s National Statement on Ethical Conduct in Human Research (2018). This study was approved by the Swinburne University of Technology Human Research Ethics Committee (reference 20214304-6249).

## Results

### Overview

A total of 7 CPs completed our usability test (Table 1); each interview took approximately 30 minutes. The mean SUS score for the system was 68.57, which indicated an above-average usability of the system. Analysis of the interview field notes revealed two major themes of concern for the participants: (1) ease of navigation and (2) minimum required information or data fields. Each of these concerns is further discussed below, including the moderator-observed and participant-reported pain points and facilitators (Textbox 1).

**Table 1:** Participants’ demographic information collected by using a self-administered questionnaire attached with the consent form.

Characteristics		Frequency, n
**Gender**		
	Male	3
	Female	4
**Age (years)**		
	20-25	1
	26-35	6
**Experience (years)**		
	1-2	1
	2-4	2
	5-10	3
	>10	1
Previously reported an ADR^a^ to a regulator, eg, TGA^b^ or SAFEVAC^c^		3

^a^ADR: adverse drug reaction.

^b^TGA: Therapeutic Goods Administration.

^c^Adverse event reporting database for Victoria.

**Observed and participant reported pain points and facilitators.**

**Barriers**

- Multiple steps when accessing or submitting the report (number of clicks)
- Unable to share with allied health (system interoperability)
- Irrelevant data fields
- Lack of system integration (web-based vs pharmacy dispensing system)
- Length (number of data fields and questions)

**Facilitators**

- Ability to search through an items list (eg, address and medications)
- Drop-down menu and auto-filled sections
- Succinct list and relevant to health care setting
- Combined use of checkboxes, drop-down menu, and free-text entry
- Direct submission of the report to the Therapeutic Goods Administration

### Ease of Navigation (System-Related Functionality)

The participants perceived the web-based digital form of ADR reporting as an efficient method. The ability to choose from the available selections in the drop-down menu, having certain data fields automatically prefilled, and the combination of categorized data entry with the option of free-text facilitated system navigation and were highly praised.

The system’s functionality was limited by the lack of clearly visible and intuitive action buttons, which complicated and slowed navigation. For instance, all participants struggled to begin the task (ie, the ADR report) when they initially logged in to the system, despite the visibility of 3 dots on the user page, which lacked clarity and instruction (Multimedia Appendix 4). In addition to this, having to navigate multiple screens after logging in before reaching the start screen was considered a pain point and potential deterrence. Participants also struggled when they needed to complete and submit the ADR report. In all interviews, the system did not allow the participants to complete and submit the ADR report due to missing data fields. The participants were confused as to why they could not complete the report, and 5 of the 7 interviewees were verbally guided by the moderator to search for any potential missing data field, which further added to the time to complete the report.

### Minimum Required Information (Data Field or Reporting Form)

#### Unnecessary Data Field Entry

All participants felt that some data entry fields could be omitted (Multimedia Appendix 5). For example, when creating a new patient participants questioned if providing “Medicare details” (personal health fund identifier) was needed by the TGA. Another participant was asked: “Is the patient’s weight really needed?” It was further stated that such a question is intrusive and could deter consumers and CPs from reporting. Some of the participants were uncertain if they could skip such sections and uncertain what information or data fields were actually pertinent for the purposes of ADR reporting.

#### Lack of Clarity (Interpretability)

Users felt that the instructions for some fields needed further clarification or simplification. For example, in the section for adding the suspected medication, the “frequency” field required clarification. Some participants were unsure about how the frequency should be interpreted, whether the options of 1 time, 2 times, or 3 times ongoing referred to the medication dose or the number of times the ADR was experienced (Multimedia Appendix 6).

#### Unnecessary Information

All participants questioned the use of specific terminologies and options, which they perceived as having no added value to the system; for example, when entering the reporter details, a participant commented: “There’s a list of various health professionals (nurse, dentist, etc; Multimedia Appendix 7), but the system is intended for community pharmacists.” Further to this, participants also commented on the list of options provided in the drop-down menu, which was not within the scope of practice and deemed irrelevant. For example, when adding the strength of the medication taken, the unit drop-down menu had multiple “irrelevant” measurements not used in everyday pharmacy practice (eg, moles, nanograms, deciliters, etc). This required the participants to scroll through a very long list of units to find the correct unit. The participants felt that this added confusion, increased time, and was also considered “distracting noise;” another participant used the phrase “death by scroll.”

## Discussion

### Overview

This paper highlights the importance of usability testing of ADR reporting systems by the target end users (CPs) and within the clinical setting in order to maximize functionality, experience, and overall interpretability of a system [30]. Poor usability may negatively impact not just user experience but also discourage ADR reporting and impact patient safety.

While interviews, focus groups, surveys, and questionnaires can provide information about user satisfaction and perceived ease of use, our study demonstrates that they can also easily overlook important information about system functionality or user interpretation. For example, comparing our above-average SUS scores (68.57) with our observational field notes, the SUS alone failed to direct attention to functional issues that could have led to misinterpretation, confusion, resistance to adoption of the system, and underreporting. By probing participants for their thoughts directly after task completion, we also gained valuable insights into CPs’ understanding of ADR reporting, including other attributes and features that may facilitate the reporting of any newly designed ADR reporting system.

### System Functionality

CPs who participated in our usability evaluation indicated that elements of the dashboard design (such as having certain data fields automatically prefilled and a combination of checkboxes, drop-down menus, and free-text entry) were perceived to be extremely useful to navigate the system and facilitated ADR reporting. The integration of auto-population features has also been identified as an efficient way to facilitate ADR reporting by CPs in previous studies [12,13,17,39].

Despite these facilitating features, we found that CPs experienced a high degree of confusion and frustration when completing ADR reports, for example, having unnecessary data field: “there’s a list of various health professionals (nurse, dentist, etc), but the system is intended for community pharmacists” (Multimedia Appendix 7). Analysis of test results highlighted that the relationship between functionality and intuitiveness of the system lacked synergy, suggesting the need for buttons to be clearly labeled in a language familiar to the intended clinical user (CPs) and contrastingly featured on the page, for example, when commencing an ADR report: “it took me a while to figure out how to start the report” (Multimedia Appendix 4). Other functionalities such as making required or missing data fields more noticeable, perhaps by highlighting the entire field or a pop-up dialogue box, can be used to assist in system interpretability and facilitate the completion of ADR reports. Health professionals already experience time constraints and may view reporting systems as an extra burden for documentation, as these systems may require extra time to access or involve duplicating tasks that offer no additional benefit to the patient [21].

Completing time-consuming activities that are irrelevant for ADR reports may cause resistance. During our observation, one CP commented “death by scroll” when searching for the appropriate unit of measurement after adding the suspected drug (Multimedia Appendix 6). Therefore, enhancing the usability of the systems can be achieved by refining the number of data fields, including the selection options, and ensuring that only the most relevant and pertinent options are available or appear first. Furthermore, fields that gather information such as concomitant therapies or product start and end dates are perceived by CPs as duplication of information that is already contained in the dispensing systems before the ADR reporting commencement. Therefore, the system would be further enhanced by integrating it directly within the CPs’ workflow. During our observation and interview, a number of the CPs mentioned that they were not aware of what information was necessary for the purposes of ADR reporting. One pharmacist commented: “I’m considering the fields with the asterisks as what’s important.” A lack of knowledge was not specifically mentioned by the CP, but these findings suggest that CPs may also benefit from more education and training on what is required for an ADR report. The need for more education and training has also been discussed in previous studies [12,13,17,23,39]. Furthermore, this is also consistent with our previous study, which explored the barriers and facilitators to ADR reporting among CPs in Australia [19].

### Lessons Learnt and Proposed Recommendations for Design of Future ADR Reporting Systems in Pharmacy and Related Health Care Settings

#### Overview

Our results are timely as health systems introduce new electronic infrastructure to improve health information sharing, such as through e-prescribing or drug information gathering [40]. If CPs are to increase their reporting of ADRs and provide high-quality information that regulators seek to capture, we must prioritize the design of reporting systems [33]. This can enable CPs to meet their care delivery goals while preventing adverse drug exposure by providing timely patient safety data. On the basis of the findings of the study, we have developed design recommendations (Textbox 2) and 4 core recommendations that may be considered in the design and implementation process when designing ADR reporting tools and systems for pharmacists in community pharmacies.

**Summary of design recommendations for health information reporting systems.**

**Design recommendations**

- Integrate reporting into existing interfaces to reduce barriers and minimize manual data entry.
- Design IT systems for clinical care that encourage information sharing and minimize duplication of work.
- Develop living documents of adverse drug reaction (ADR) reports, enabling multiple providers to edit, update, and remove data as needed.
- Link ADR reporting to clinical documentation to prevent harmful reexposures.
- Include only relevant data fields for clinical practice in reporting systems.
- Enable standardized and categorized data entry with free-text options.

#### Integrated Reporting Systems

We see an opportunity to integrate ADR reporting into existing dispensing systems. This can be enhanced by incorporating reporting into the clinical workflow. If data fields can also be auto-populated with readily available information, this can avoid duplicate data entry. Further to this, reducing the time (eg, entering multiple passwords) to access reporting platforms can also be minimized.

#### Combined Checkboxes Drop-Down Menu and Free-Text Entry

To speed up reporting, systems should enable the combination of standardized and categorized data entry and drop-down menu while allowing free-text entry in other locations so CPs can document nuanced information for complex events or reactions.

#### Data Sharing With Care Providers and Consumers

In addition to providing safety data to regulators, ADR reporting systems must act as a mechanism to document work and share information between allied health providers and consumers. This may be enhanced by enabling multiple providers to update, edit, and remove data as new information becomes available or when a patient’s condition changes. It may also be useful to develop infrastructure to share data with consumers and empower consumer reporting.

#### Relevant Data Fields

Developers need to consider how confusion over the intent of data fields might impact the use of reporting systems. Therefore, reporting systems that CPs are expected to use should only include data fields and information relevant to clinical practice and PV to minimize confusion and frustration.

### Limitations

Direct observations may have influenced participants’ behavior and responses. The use of a think-aloud moderating technique may have disrupted the natural thought process of our participants. The study did not analyze the time it took to complete a report, which may have influenced the SUS scores. The study includes a small sample of CPs in Victoria, Australia. Finally, while the study was configured to represent clinical practice, it was conducted in a test environment, so the results may not be generalizable to a broader population of CPs.

### Conclusions and Future Work

Our study suggests that CPs are interested in reporting ADRs and would welcome reporting mechanisms that meet clinical needs while allowing them to provide patient safety information. Usability studies with direct observation provide crucial insights into CPs’ interactions with ADR reporting systems, identifying nuanced barriers and “pain points” often missed by surveys or quantitative measures like the SUS. Demonstrating the value of usability testing, our findings underscore its significance in evaluating and refining newly designed health information systems before implementation in primary care settings. Building on the significance of this study, it stands as the first usability evaluation of an ADR reporting system with CPs in Australia, which warrants further refinement and increased awareness [17]. Exploring ADR reporting systems within the pharmacy practice setting offers a promising avenue for future investigations [17,19]. Investigating the minimum required data set for CPs to document and communicate ADRs also presents a valuable area of future research.

## Appendix

Multimedia Appendix 1 Map of adverse drug reaction (ADR) report from the GuildCare system to the Therapeutic Goods Administration (TGA).

Multimedia Appendix 2 System Usability Scale (SUS) calculation.

Multimedia Appendix 3 System Usability Scale (SUS) Scale.

Multimedia Appendix 4 Example screenshot of the main screen to select and start the adverse event report.

Multimedia Appendix 5 An example screenshot of the screen to add suspected medication displayed by the frequency (left) and option available (right).

Multimedia Appendix 6 An example screenshot of the screen when participants begin to report an adverse drug reaction (ADR), displaying the option to add patients’ weight.

Multimedia Appendix 7 An example screenshot of the screen to add the reporter details and the options available under reporter type.

## Abbreviations

ADR: adverse drug reaction
CP: community pharmacist
PV: pharmacovigilance
SUS: System Usability Scale
TGA: Therapeutic Goods Administration
WHO: World Health Organization

## Data Availability

All data generated or analyzed during this study are included in this published article (and its supplementary information files).
