# Supplementary figures and images for: A Web-Based Tool to Report Adverse Drug Reactions by Community Pharmacists in Australia: Usability Testing Study
Source: JMIR Form Res. 2023 Sep 29;7:e48976. doi: 10.2196/48976 (PMC10576234; doi:10.2196/48976)

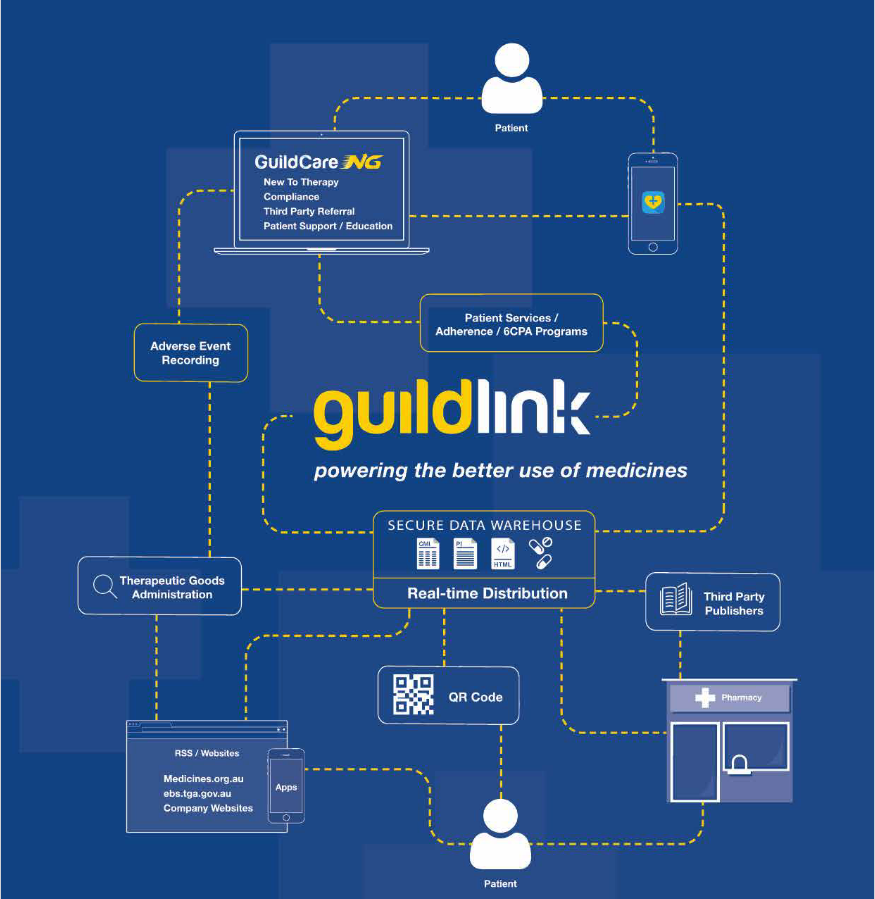

Supplement: Multimedia Appendix 1 [file formative_v7i1e48976_app1.png]

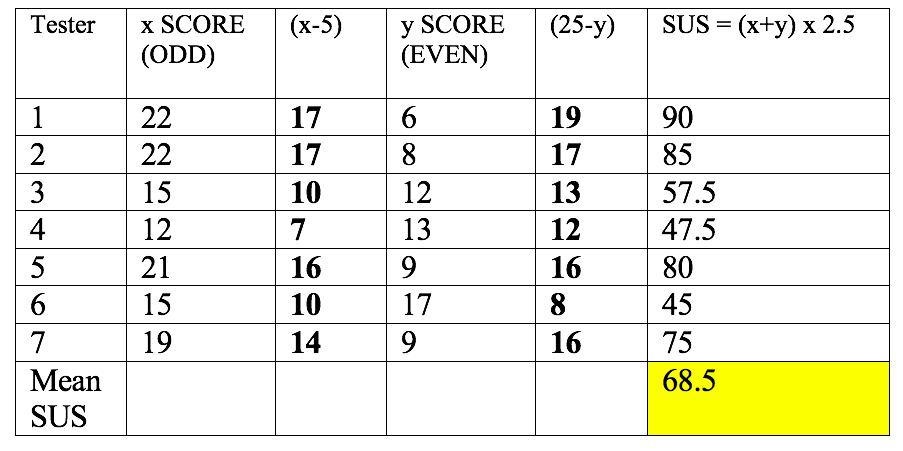

Supplement: Multimedia Appendix 2 [file formative_v7i1e48976_app2.png]

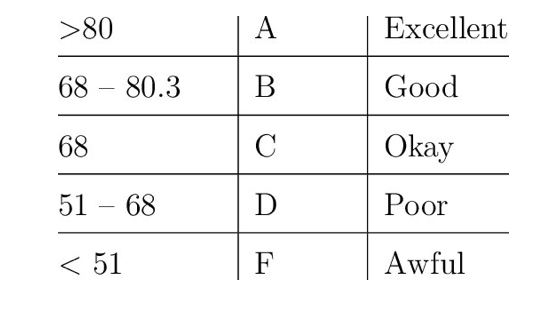

Supplement: Multimedia Appendix 3 [file formative_v7i1e48976_app3.png]

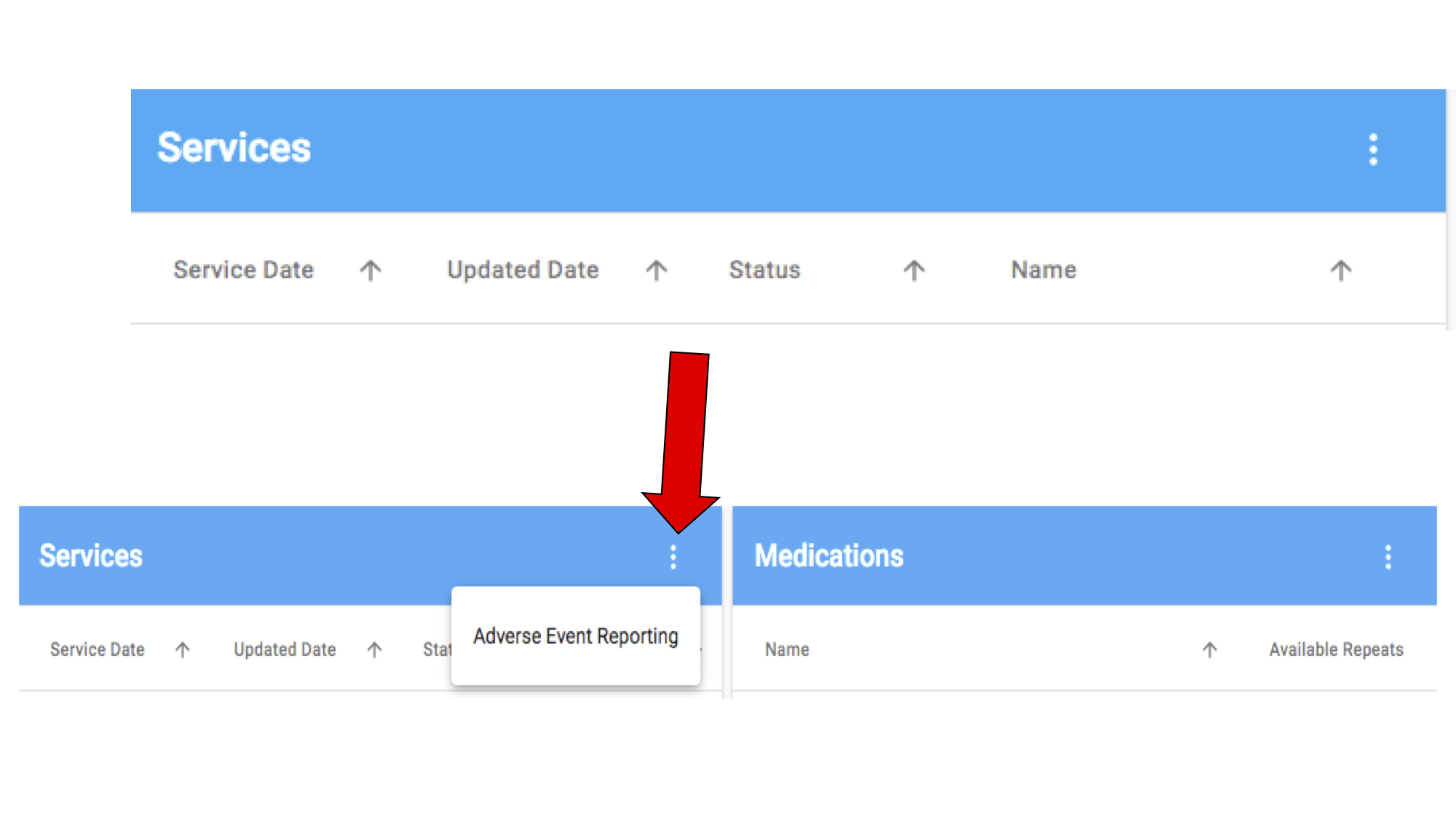

Supplement: Multimedia Appendix 4 [file formative_v7i1e48976_app4.png]

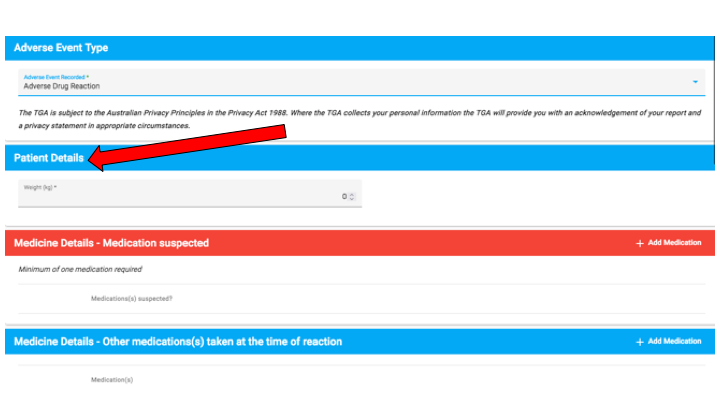

Supplement: Multimedia Appendix 5 [file formative_v7i1e48976_app5.png]

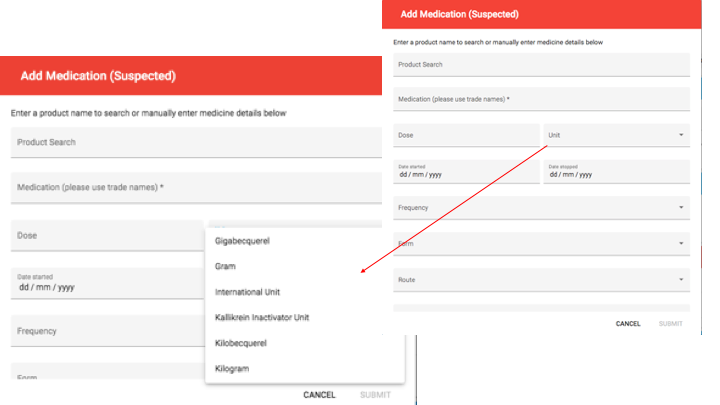

Supplement: Multimedia Appendix 6 [file formative_v7i1e48976_app6.png]

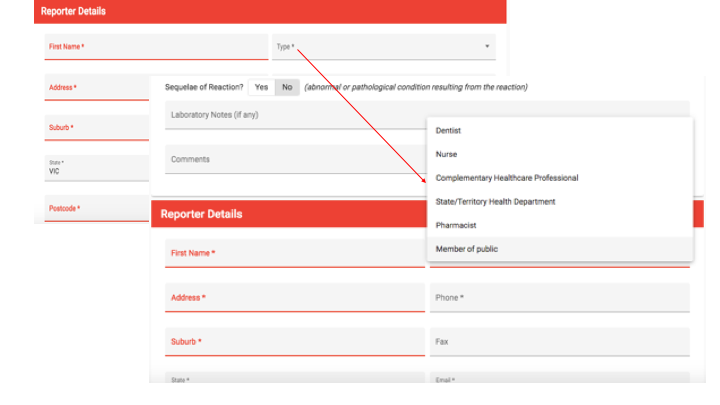

Supplement: Multimedia Appendix 7 [file formative_v7i1e48976_app7.png]
